# A Novel Bipartite Nuclear Localization Signal Guides BPM1 Protein to Nucleolus Suggesting Its Cullin3 Independent Function

**DOI:** 10.1371/journal.pone.0051184

**Published:** 2012-12-10

**Authors:** Dunja Leljak Levanić, Tomislav Horvat, Jelena Martinčić, Nataša Bauer

**Affiliations:** Department of Molecular Biology, Faculty of Science, University of Zagreb, Zagreb, Croatia; Semmelweis University, Hungary

## Abstract

BPM1 belongs to the MATH-BTB family of proteins, which act as substrate-binding adaptors for the Cullin3-based E3 ubiquitin ligase. MATH-BTB proteins associate with Cullin3 via the BTB domain and with the substrate protein via the MATH domain. Few BPM1-interacting proteins with different functions are recognized, however, specific roles of BPM1, depending on its cellular localization have not been studied so far. Here, we found a novel bipartite nuclear localization signal at the C-terminus of the BPM1 protein, responsible for its nuclear and nucleolar localization and sufficient to drive the green fluorescent protein and cytoplasmic BPM4 protein into the nucleus. Co-localization analysis in live *Nicotiana tabacum* BY2 cells indicates a Cullin3 independent function since BPM1 localization is predominantly nucleolar and thus devoid of Cullin3. Treatment of BY2 cells with the proteasome inhibitor MG132 blocks BPM1 and Cullin3 degradation, suggesting turnover of both proteins through the ubiquitin–proteasome pathway. Possible roles of BPM1 in relation to its *in vivo* localization are discussed.

## Introduction

The eukaryotic cell is highly compartmentalized, and correct protein localization is required for its proper function [1]. Two large compartments, the nucleus and the cytoplasm, wherein proteins are synthesized, are separated by the nuclear envelope. Proteins play a major role in most cellular processes but must be appropriately located in order to fulfill their functions. It is well documented that single eukaryotic genes can give rise to proteins that are targeted to several subcellular locations. Differential distribution may be achieved if two or more translation products that either harbor or lack specific localization signals are synthesized in the cell, or if the targeting signal becomes inaccessible to a certain subpopulation of the same protein. Accessibility of the localization signal could be controlled by protein folding, hindrance by other proteins, or post-translational protein modification [2]. Proteins involved in chromosomal stability, replication, gene transcription, RNA processing, ribosome subunit assembly, cell cycle regulation *etc*. are subject to both active nuclear import and export driven by specific nuclear localization signals (NLS) or nuclear export signals (NES). The classical nuclear import pathway involves the NLS containing short stretches of lysine or arginine residues and is recognized by proteins belonging to the importin super-family (importin α and β), which allow transport across the nuclear envelope through Ran-GTP [3]. Several nuclear export pathways have been identified and the best known involves the NES, which consists of short canonical sequences rich in hydrophobic amino acids, often leucine and isoleucine.

Although a high number of proteins with the *B*ric-a-Brac, *T*ramtrack, *B*road Complex (BTB) domain have been discovered in plants within the past decade [4], [5], little is known about their function. The BTB domain, also known as the POZ domain, is a protein-protein interaction motif which enables dimerization and oligomerization, as well as interactions with a number of other proteins. It was shown for some BTB proteins that, to accomplish their proper function they occasionally have to alter subcelular localizatioin. For example, during normal plant growth, BTB-containing NPR1 is present as an oligomer in the cytosol, but upon salicylic acid activation it is partially reduced to a monomeric form and imported into the nucleus, where it regulates the expression of pathogenesis-related genes [6]. BTB-containing proteins are abundant in animals and control a variety of unrelated but fundamental cellular processes ranging from chromatin conformation and actin dynamics to cell cycle regulation. The disturbance in activity of many BTB proteins often results in various diseases, including cancer [7]. As in animals, the BTB proteins are also abundant in plants and in *Arabidopsis thaliana* there are 80 members of the BTB superfamily. Unfortunately, much less is known about their roles in plant development. However, in spite of the high sequence divergence and apparently unrelated functions, many BTB-containing proteins have at least one common role: recruitment of target substrates to E3 ubiquitin ligase complexes [8]. E3 ligases attach ubiquitin to target proteins and are critical components in the ubiquitin dependant protein proteolysis by the 26 S proteasome complex. The protein family contains a BTB/POZ domain located at the C-terminus and a *M*eprin *a*nd *T*RAF *H*omology (MATH) domain located close to N-terminus. The MATH-BTB domain architecture emerged in multicellular eukaryotes with organism-specific frequency [5]. In the *A. thaliana* genome 6 *BPM* genes are present. Owing to the BTB domain, BPM proteins are capable of forming homo- and heterodimers and can assemble with Cullin3A (CUL3A) and CUL3B [9]. These in turn take part in formation of CUL-dependant E3 ubiquitin protein ligase complexes, with the assembly mechanism conserved in animals and plants [10]. Substrate ubiquitination is dependent upon CUL3-BTB domain interaction while substrate recognition is mediated by the MATH domain. In animals, MATH-BTB containing proteins interact with different substrates, like the katanin AAA-type ATPase protein MEI-1 [10], [11], Ci/Gli2/Gli3 transcription factors [12], the polycomb protein BMI1 and the histone MacroH2A [13]. Such boundless repertoire of target proteins might be provided by the diversification within the MATH domain. A high level of diversification within the rapidly evolving family of MATH-BTB proteins in rice [14] is in contrast with the remarkably conserved *A. thaliana* MATH-BTB proteins, indicating potentially different downstream strategies in regulating *Arabidopsis* MATH-BTB functions, such as in alternative splicing or intracellular trafficking.

As subunits of multimeric CUL3 ubiquitin ligase complexes, MATH-BTB proteins can mediate and modulate ubiquitination and, in doing so, regulate diverse biological processes like development, cell cycle and response to pathogens. Ubiquitin ligases covalently bind ubiquitin to specific protein substrates and mediate ubiquitination as an important and functionally-related posttranslational modification of proteins or polyubiquitinate proteins and mark them for subsequent degradation by the 26 S proteasome [15]. Alternatively, it is possible that MATH-BTB proteins bind various substrate proteins in an ubiquitination-independent manner, modulating their action and localization.

We were curious about the sub-cellular localization of BPM1 and its dependence on CUL3 ubiquitin ligase related activities. To assess the spatial activity of BPM1 we examined how BPM1 is driven into the nucleus, what additional functions it may have and whether it is regulated by proteasomal degradation machinery.

## Results

### Subcellular Localization of BPM1

**Figure 1:**
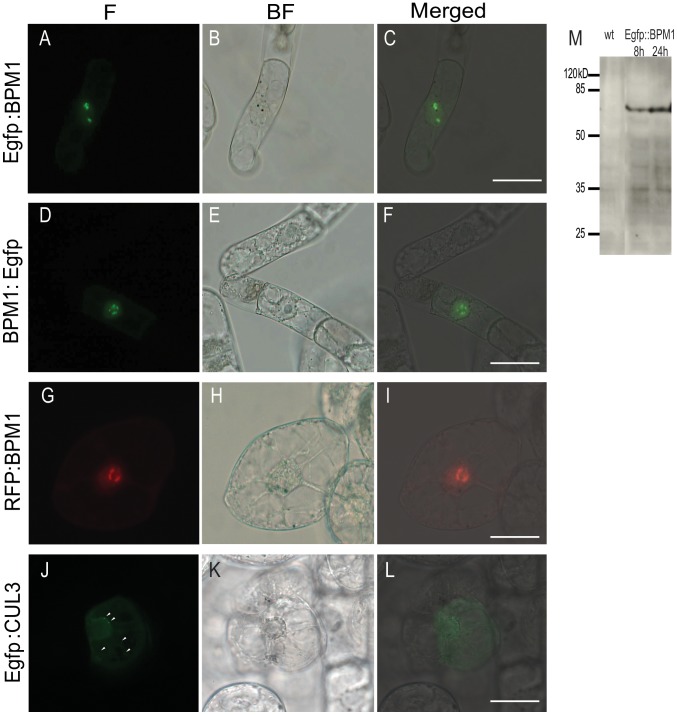
Subcellular localization of BPM1 and CUL3 proteins in the tobacco BY2 cells. Intact BPM1 protein (Egfp:BPM1, BPM1:Egfp and RFP:BPM1) is dispersed throughout the cytoplasm, forming agglomerates in the nucleus (A–I). CUL3 is dispersed in both the cytoplasm and the nucleus, additionally forming cytoplasmic speckles (arrowhead) (J–L). M: Detection of the complete Egfp:BPM1 protein (73 kD) from BY2 cells by western blot analysis, 8 and 24 hours after biolistic transformation. Images shown are bright field (BF), Egfp and RFP fluorescence (F), and a merged image. Bars are 50 µm.

Various utilized protein localization prediction software tools revealed inconsistent results, mostly predicting BPM1 as a chloroplastic and/or mitochondrial protein. Only Plant-mPLoc software predicted BPM1 placement at the cellular membrane and in the nucleus. For subcellular localization analysis C- and N-terminal BPM1-fluorescent protein fusions were made (Egfp:BPM1, BPM1:Egfp and RFP:BPM1), and their localization followed *in vivo* in *N. tabacum* BY2 cells (Fig. 1A–I). In some cells low amounts of BPM1 were detected in cytoplasm, whereas the accumulated protein formed noticeable agglomerates in the nuclei of all cells. Full-length Egfp:BPM1 fusion protein could be confirmed in the transformed cells by western blot analysis (Fig. 1M) as long as the fluorescent signal was detectable.

**Figure 2:**
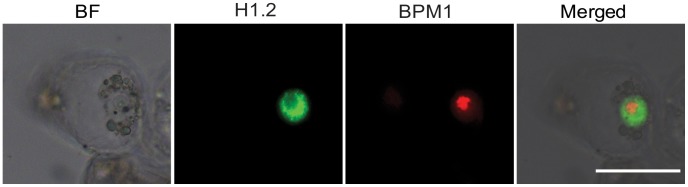
Subcellular co-localization of histone H1.2 (Egfp:H1.2) and BPM1 (RFP:BPM1) in the tobacco BY2 cells. The BPM1 predominantly forms agglomerates in the nucleolus. BF: bright field. Bar is 50 µm.

In order to precisely define the BPM1 subnuclear localization we co-transformed BY2 cells with Egfp:H1.2 and RFP:BPM1. Linker histone H1.2 is a general visualization marker of the nucleoplasm. It is uniformly distributed within the cell nucleus and not in the nucleoli [16]. In BY2 cells co-transformed with histone H1.2 and BPM1 a predominant nucleolar along with dispersed nucleoplasmic BPM1 localization was obvious (Fig. 2).

**Figure 3:**
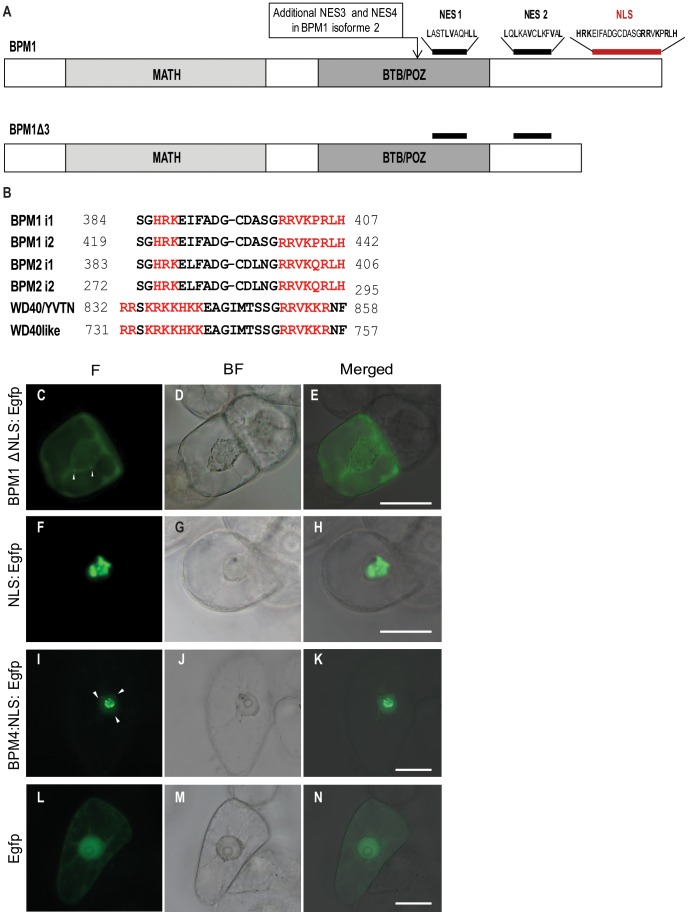
Identification of NES and NLS in the *A. thaliana* BPM1 protein sequence and subcellular localization of BPM proteins and Egfp in the tobacco BY2 cells. Schematic representation of the BPM1 protein containing two NESs (4 NESs are present in isoform 2) and one NLS signals, and of BPM1Δ3 protein devoid of NLS (A). Nucleotide sequence alignment of BPM1 NLS (AED92637.1) with similar sequences: BPM1 i2: AED92638.1, BPM2 i1: Q9M8J9.1, BPM2 i2: AEE74359.1, WD40/YVTN: AED95810.1; WD40like: BAB09913.1 (B). BPM1 protein devoid of NLS localizes to the cytoplasm (BPM1Δ3:Egfp; C–E). The C-terminal NLS sequence of BPM1 is sufficient to import GFP (NLS:Egfp; F–H), as well as the BPM4 protein (BPM:NLS:Egfp; I–K) into the nucleus. Control BY2 cells were transformed with GFP alone (Egfp; L–N). Images shown are bright field (BF), Egfp fluorescence (F), and a merged image. Arrowheads indicate cytoplasmic agglomerates of a particular protein. Bars are 50 µm.

PredictProtein server software failed to identify an NLS in the BPM1 protein sequence, whereas the software NetNES predicted two NESs in BPM1 isoform 1 (leucine position L106 and L332) and 4 NES signals in BPM1 isoform 2 (leucine position L261, L263, L264 and L367). However, based on detailed description of NES and NLS sequences given by Sekimoto et al. [17] we found one NLS and two leucine-rich NESs in BPM1 isoform 1 sequence (Fig. 3A). Classical NLS sequences generally appear either as a single-stretch or as two small clusters of basic residues separated by approximately 10 amino acid residues, with the respective consensus sequences of the monopartite and bipartite basic NLS’s (K/R)4–6 and (K/R)2 X10–12(K/R)3. We found a bipartite NLS protein sequence HRKEIFADGCDASGRRVKPRLH with the two typical short stretches of positively charged amino acids (underlined) spaced by 11 amino acids at the C-terminal end of BPM1 (Fig. 3A). According to the sequence analysis, similar NLS sequences exist at the C-terminus of both *A. thaliana* BPM2 isoforms and in the WD40/YVTN repeat and Bromo-WDR9-I-like domain-containing proteins (Fig. 3B).

NES sequences are often rich in hydrophobic amino acids, especially leucine and isoleucine. Based on the leucine-rich consensus sequence, we predicted two NESs in the BPM1 protein (aa 279–289 and aa 330–346, Fig. 3A). BPM1 isoform 2 differs from the canonical isoform 1 by additional 35 amino acids (F254 → FKVLPLTLLLIVYSRMYHPGSSPGALLLFSSLLTRD). This peptide contains two additional putative NESs (NES3 and NES4; underlined above). All NES positions were confirmed with NetNES software, except for NES1 (L106), which was eliminated due to the presence of charged flanking amino acids [17].

The predicted NLS from BPM1 is responsible for nuclear and nucleolar targeting.

To analyze whether the supposed NLS of BPM1 protein is truly responsible for nuclear localization, we transformed BY2 cells with the truncated BPM1 gene devoid of 30 amino acids from the C-terminus (*BPM1Δ3*, Fig 3A), tagged with GFP at the N- and C- terminus. The truncated BPM1 protein localized to the cytoplasm, where it sporadically formed agglomerates, failing to accumulate in the nucleus (Fig. 3C–E). Furthermore, we tested the ability of the predicted NLS to drive an exogenous protein into the nucleus. Uniform sub-cellular localization of Egfp in the cytoplasm and the nucleus (Fig. 3L–N) was altered following the fusion of the predicted NLS to Egfp, leading to accumulation of the recombinant protein in the nucleus (Fig. 3F–H). Similarly, fusing the predicted NLS to the C-terminus of cytoplasmic BPM4 [18] resulted in nuclear protein accumulation, predominantly in the nucleolus, in the form of agglomerates similar to those seen with the BPM1 protein (Fig. 3I–K). Small cytoplasmic fractions of BPM4:NLS:Egfp were subject to agglomeration as well.

### Nucleolar BPM1 is Deprived of CUL3

It was previously shown that the members of the MATH-BTB protein family from *Arabidopsis* and rice can interact with CUL3 proteins [14], [19] and thus likely act as substrate-specific adaptors in CUL3-based E3 ubiquitin ligase. Due to toxicity of *A. thaliana* CUL3 for bacterial cells, which suppressed bacterial growth preventing plasmid multiplication that would be sufficient for biolistic transformation, the available vector containing *Z. mays* CUL3 was used. An alignment of CUL3 protein sequences from the two organisms [20] indicated a high degree of conservation (78% identity).

**Figure 4:**
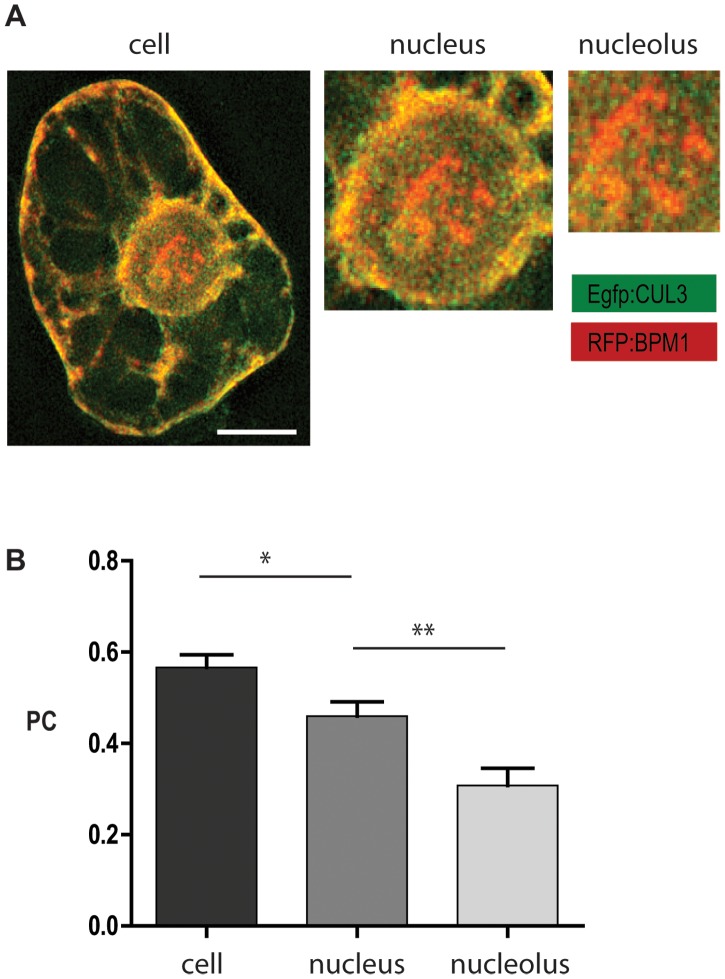
Co-localization analysis of BPM1 and CUL3 in the tobacco BY2 cells. BPM1 and CUL3 signals display a complete overlay at the level of the entire cell, including the nucleus and nucleolus (A). Pearson’s coefficient (PC) reflects a reduction in protein co-localization level in the nucleus and nucleolus (B). N = 18. Bar is 10 µm.

Single biolistic transformation of BY2 cells with CUL3, as well as dual-color fluorescent imaging following co-transformation with RFP:BPM1 and Egfp:CUL3 resulted in protein co-localization both in the cytoplasm, along cytoplasmic strands, and in the nucleus. However, CUL3 signals were weak and dispersed sporadically with small bright dots in cytoplasm, whereas BPM1 signals reflected distinguishable agglomerates in and around the nucleolus (Fig. 1J–L, Fig. 4A). To investigate CUL3 and BPM1 protein co-localization in different cellular compartments three-dimensional space stacks of deconvolved images were analyzed separately, considering the whole cell, as well as the nucleus and nucleolus in particular. Statistical analysis of measured Pearson’s coefficient (PC) values clearly demonstrated the highest co-localization when the whole cell was considered (Fig. 4B), reflecting even distribution of both proteins mostly in the cytoplasm. Interestingly, significantly lower mean PC values were obtained by analyzing the nucleus and the nucleolus, the latter being devoid of CUL3 to a large extent (Fig 4B).

### BPM1 and CUL3 are Degraded by the 26 S Proteasome

**Figure 5:**
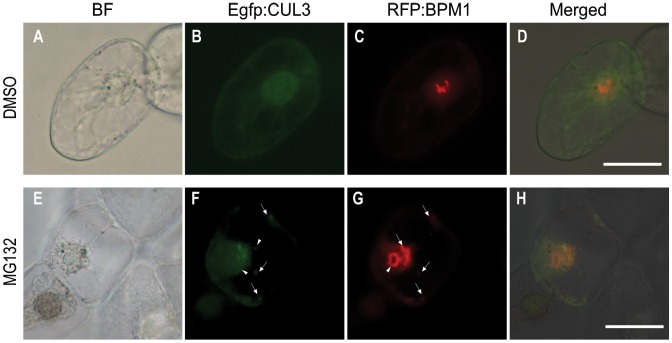
Subcellular localization of CUL3 and BPM1 proteins in the tobacco BY2 cells. CUL3 and BPM1 overlap in the cytoplasm but not in the nucleolus (A–D). After proteasome inhibition with 50 µM MG132, CUL3 and BPM1 accumulate in the cytoplasm which implies their cytoplasmic proteosomal degradation (E–H). Images shown are bright field (BF), Egfp and RFP fluorescence from CUL3 and BPM1 respectively, and a merged image. Arrowheads indicate cytoplasmic agglomerates where both proteins co-localize. Arrows indicate agglomerates of a particular protein. Bars are 50 µm.

Monitoring BPM1 and CUL3 fluorescent signals in more than 400 cells confirmed their appearance a few hours after bombardment, with the maximum intensity eight hours after. Subsequently, signals started to vanish until complete disappearance 2 days after biolistic transformation. Following the treatment of BY2 cells with the 26 S proteasome inhibitor MG132 (50 µM), periodical monitoring revealed the presence of Egfp/RFP labeled proteins even after 4 days. Interestingly, localization of BPM1 and CUL3 in co-transformed BY-2 cells treated with MG132 was altered and different than localization of proteins in untreated or DMSO treated controls (Fig. 5). In addition to usual uniform CUL3 signal in untreated cells, many bright dots that only partially co-localized with BPM1 were observed after MG132 treatment. Moreover, besides dominant nucleolar accumulation following MG132 treatment, BPM1 also aggregated in the cytoplasm and around the nucleus (Fig. 5E–H). Increased cytoplasmic accumulation of BPM1 and CUL3 in cells where proteasomal degradation was inhibited by MG132 treatment, indicates that both proteins are degraded in the cytoplasm in a proteasome dependant manner. Accordingly, following the MG132 treatment, the nucleolar BPM1 localization stayed intact even after 4 days.

## Discussion

Our results regarding subcellular localization of BPM1 confirm previous observations, wherein BPM1 localized to the nucleus of both *N. benthamiana* leaf epidermal cells [18] and the BY2 cells [21]. BY2 cells can be easily transformed and are a powerful tool widely used for exploring the molecular and cellular biology of plant cells. Determining the sub-cellular localization of a protein within a cell is often an essential step towards understanding its function. Here, we show a weak dispersed cytoplasmic signal and a nuclear signal with abundant BPM1 agglomerates in the nucleolus.

The nucleolus is the most obvious nuclear sub-compartment. Even though its primary role is ribosome biogenesis, emerging evidence substantiate its role in numerous non-ribosomal functions as well [22]. Recently, it was described that the inactivated human X chromosome, as well as autosomes carrying the ectopic X-inactivation center sequences, are targeted to the nucleolus during mid-to-late S phase [23]. Interestingly, the inactive X chromosome and heterochromatic regions in the genome are enriched with a histone variant macroH2A, known to play a role in gene repression, control of cell cycle and cell proliferation [24]. Described interaction of the SPOP protein, a MATH-BTB homolog of BPM1 in humans, with the histone macroH2A [25], [26] suggests a possible role for BPM1 in epigenetic mechanisms taking place in the nucleolus as well, even though there is no macroH2A homolog in the plant genome.

Multiple roles for BTB-containing proteins were previously described. These include control of chromatin structure, transcriptional regulation [27], [28] http://www.ncbi.nlm.nih.gov/pubmed/1985916, protein degradation [9], [10], [11], [29], cytoskeleton organization [30], [31] and ion transport [32]. Apart from interactions regulated by the BTB domain, MATH-mediated BPM1 interactions with different cytoplasmic and nuclear proteins were described recently, which further emphasizes various BPM1 roles based on sub-cellular localization. Screening the root-specific Y2H cDNA library using the BPM1 MATH domain, resulted in the identification of different protein interactors [33]. These include several transcription factors: ERF/AP2 (*Ethylene Response Factor/APETALA2*), RAP2.4 [18], MYB56, which belongs to R2R3-MYB protein family and is involved in stress response and establishment of plant cells identities and fates [34], [35], and an ABA-responsive NAC transcription factor VNI2, which integrates plant responses to environmental stress by modulation of leaf longevity [36]. Other interactors are the multifunctional protein DDB1a located in the cytoplasm and nucleus, acting as a substrate adaptor for CUL4 E3 ubiquitin ligase [37], [38] and playing a role in epigenetic regulation of gene expression [39], LEA4 protein, which plays an important role in water stress tolerance [40], a serine/threonine protein kinase and some proteins of unknown function. In addition, Lechner et al. [21] demonstrate that BPM proteins interact directly with the ATHB6 transcription factor, which is a negative regulator of ABA responses, and regulate its degradation via the CUL3 E3 ubiquitin ligase.

Gingerich et al. [14] predict that most plant BTB proteins are able to act as CUL3 E3 ubiquitin ligase target adaptors. However, when previously published data from Y2H screens and pull down assays is taken into consideration, this prediction is not always experimentally substantiated. Weber et al. [9] demonstrated that BPM1 and BPM3, unlike BPM5 and BPM6, interact with CUL3A and CUL3B while in Figueroa et al. [41] BPM1, BPM2, BPM3 and BPM6 all interacted with CUL3A. Moreover, the *in vitro* assays are rather limited since they do not reflect *in vivo* circumstances that are preconditions for interaction such as certainty of protein co-localization.

Here, the spatial overlap between BPM1 and CUL3 *in vivo* is presented for the first time. We observed differences in sub-cellular localization of BPM1 and CUL3 proteins. In BY2 cells, CUL3 exhibited cytoplasmic and dispersed nuclear localization, which is in agreement with cytoplasmic CUL3 localization in animal HEC293 cells [42]. Moreover CUL3 in our experiment was mainly excluded from the nucleolus, the major localization site for BPM1, in which those two proteins did not overlap. Since both fusion proteins were expressed under the constitutive 35 S promoter, a possibility of false localization due to the protein overaccumulation in the heterologous cell system has to be considered. Recently, it was demonstrated that the majority of the CUL3 proteins exist as dimers or multimers in cells. Formation of such CUL3 dimers and formation of a functional E3 ligase complex is dependent on dimerisation of CUL3 substrate adaptors (MATH-BTB or Kelch-BTB proteins) via the BTB domain [42]. According to these results, one would expect that overexpression of co-transformed BPM1 and CUL3 would lead to their stronger co-localization driven by higher potential for dimerisation and E3 ligase complex formation, which was not the case in our experiments. Consequently, we strongly believe and suggest that in nucleolus BPM1 has a function unrelated to CUL3.

Therefore, co-localization analyses imply various and potentially unrelated functions of BPM1 and CUL3. Despite the Y2H and *in vitro* studies demonstrating BPM1 and CUL3 interaction [9], [19], [41], based on our analysis that revealed the lowest level of protein co-localization in the nucleolus, we presume that in this sub-compartment BPM1 could have a role different than protein ubiquitination via CUL3 E3 ligase.

BPM1 likely has various physiological and developmental roles. In this context we propose at least three different modes of BPM1 action. In the cytoplasm, BPM1 could assemble with CUL3 E3 ubiquitin ligase and mediate 26 S proteasome protein degradation, while in the nucleus, BPM1 could be involved in regulation of transcription by binding to transcription regulators [18], [21]. The nucleolar fraction of BPM1 identified here suggests an additional set of functions. In the preliminary experiment (tandem affinity purification/liquid chromatography/mass spectrometry) we identified histone H4 and histone variant H2A.Z (HTA11) as BPM1 interactors (data not shown), suggesting the role of nuclear/nucleolar BPM1 protein in the regulation of chromatin structure.

Diverse functions of a particular protein in different cellular compartments, as reviewed in Karniely and Pines [2], could be regulated by different stimuli, such as change in cellular reduction potential or phosphorylation [6], [43], [44]. We provided evidence that BPM1 contains a functional NLS sequence driving it into the nucleus. We also identified very similar bipartite NLSs in BPM2 and WD40/YVTN repeat and Bromo-WDR9-I-like domain-containing proteins, whereas it shared less homology with the NLS we identified at the very end of the BPM3 protein. Although proteins often contain more than one NLS sequence [45] we could not predict any additional ones in BPM1. Instead, we show that the deletion of the predicted NLS sequence resulted in failure to drive the protein into the nucleus and absence of nucleolar accumulation. Moreover, the unique NLS fused to a non-nuclear protein BPM4 and Egfp, was sufficient to guide them to the nucleus. The NLS tagged BPM4 exhibited a pattern of nucleolar localization similar to that of BPM1, which was particularly impressive. However, the question remains whether the mistargeted nucleolar BPM4 (or other members of BPM family) could assume similar functions in mediating biological processes only by interacting with the same substrates as BPM1, and localize to the nucleolus through those substrates, or if the detected NLS *per se* is sufficient for nucleolar localization.

In conclusion, the C-terminal NLS sequence is sufficient to guide nuclear localization of BPM1. Conversely, a pair of helices adjacent to the BTB domain at the C-terminal part of various proteins is responsible for their interaction with CUL3 [26]. Since these helices are close to the NLS in BPM1, the complex formation between BPM1 and CUL3 could potentially interfere with the NLS signal, thus blocking BPM1 targeting into the nucleus. On the other hand, the C-terminal positioning of NLS in the BPM1 sequence enables the exposure and easier access to importin proteins, implicated in nuclear targeting. In addition, it increases the possibility of producing different variants, including even those without an NLS, by alternative splicing or posttranslational processing. If this is the case, elucidation of an NLS motif will facilitate further investigations of the sub-cellular trafficking and biological roles of BPM1.

Following MG132 treatment, BPM1 and CUL3 proteins accumulated in the cytoplasm and remained there longer than in non-treated cells. This suggests cytoplasmic proteasomal degradation of both proteins, but the issue regarding different compartmentalization, regulation of function and degradation during plant growth and development remains to be examined. One of the proposed mechanisms includes protein phosphorylation based on the existence of multiple serine, threonine and tyrosine residues at the N-terminal part of BPM1 protein, as well as in the proximity of putative substrate binding sites and NESs and NLS signals. In addition, the BPM1 sequence contains an N-terminal 126DSGPYT131 motif, similar to the canonical phosphodegron DSGxxS. Phosphorylation of either phosphodegron controls the level of the protein *in vivo* and is sufficient to recruit SCF (βTrCP) and initiate the ubiquitin-mediated degradation [46], [47]. The existence of DSGxxT phosphodegron in the BPM1 sequence indicates its potential degradation via the CUL1 pathway. Altogether, specific sub-cellular interaction of BPM1 with CUL3 and its proteasome dependant degradation (possibly by CUL1), places BPM1 inside a complex interplay of different E3 ubiquitine ligases responsible for regulation of a variety of cellular processes.

The role of protein modification and subcellular localization in determination of BPM1 function remains to be elucidated. In this respect, we hope regeneration of transgenic plants overexpressing the BPM1 protein, which we recently obtained in our Lab, will help to resolve many unanswered questions.

## Materials and Methods

### Plasmid Construction

The plasmids were constructed using Gateway Technology (Invitrogen, www.invitrogen.com). The PCR fragments were cloned into pDONOR207 and sequenced (Macrogen, Korea). The coding region of *BPM1* (AtG519000.1) was amplified from U24902 stock (TAIR) using the forward (BPM1fw: ACAAGTTTGTACAAAAAAGCAGGCTCCATGGGCACAACTAGGGTC) and reverse (BPM1rev: ACCACTTTGTACAAGAAAGCTGGGTAGTGCAACCGGGGCTTCAC) primers. The BPM1 gene was shuttled from an entry clone into different destination vectors [48] as follows: pB7FWG2,0 for obtaining construct further labeled as BPM1:Egfp; pB7WGR2,0 for obtaining RFP:BPM1 construct and pB7WGF2,0 for obtaining Egfp:BPM1.

A truncated version of *BPM1* gene with the deleted NLS, further labeled as *BPM1Δ3* (Fig. 3A), was amplified using BPM1fw and BPM1delta3rev (ACCACTTTGTACAAGAAAGCTGGGcttagcctcgccacatactgc) primers and shuttled to the pB7FWG2,0 destination vector, giving the construct BPM1Δ3:Egfp and to the pB7WGF2,0 for obtaining Egfp:BPM1Δ3.

Nuclear localization signal from the 3′ end of the *BPM1* gene was amplified using NLSbpm1fw (ACAAGTTTGTACAAAAAAGCAGGCTCCGGATCCGCAATTAAGCTTATGGCGAGGCTAAGTGAACACTC) and BPM1rev primers and shuttled to the pB7FWG2,0 destination vector for obtaining NLS:Egfp.

The *BPM4* gene was amplified using cDNA from 3 day old *A. thaliana* seedlings as template along with BPM4fw (CCGGATCCATGAAATCTGTCATTTTCACAGAG) and BPM4rev (GGAAGCTTTCCATCTTCTAGTTCTGCCATTGG) primers. The amplified *BPM4* gene, as well as the entry clone containing the *NLS* sequence, were cut with *Bam*HI and *Hind*III restriction enzymes (underlined in BPM4 forward and reverse, and NLSbpm1fw primer sequences). After ligation, the entry clone with *BPM4* fused to *NLS* was reconstructed. The gene was shuttled from the entry clone to the pB7FWG2,0 destination vector for obtaining BPM4:NLS:Egfp.

For nucleus visualization we used the H1.2 linker histone (construct Egfp:H1.2; [49]).

The *CUL3* sequence was shuttled to the pB7WGF2,0 vector for obtaining Egfp:CUL3, the details on the *CUL3* cloning are reported in Juranić et al. (31).

The vector pMON30049 (Egfp, [50]) was used as a positive control for transient transformation experiments.

### Transient Transformation of BY2 Cells

BY2 cells (PC-1181 *Nicotiana tabaccum* L. cv. Bright Yellow 2) can be ordered at the Leibniz Institute DSMZ-German Collection of Microorganisms and Cell Cultures (http://www.dsmz.de). Cells were transiently transformed by particle bombardment using plasmids encoding Egfp- and/or RFP- fusion proteins under the control of the constitutive 35S promoter. To prepare plasmid-coated gold particles, 5–10 µg of plasmid solution (0.5 µg/µl) was added to 50 µl aliquots of 20 mg/ml gold suspension (0.4–1.2 µm, Heraeus). Fifty µl of 2.5 M CaCl_2_ and 20 µl of 0.1 M spermidine were added and DNA-coated particles were washed 3 x with 100% ethanol. Cells were bombarded with 7.5 µl aliquots of plasmid-coated gold particles, using the particle delivery system PDS1000/He (BioRad), 1,100-psi rupture discs, a partial vacuum of 26 inch Hg, and a 9 cm target distance. *N. tabaccum* BY2 cell line was maintained as described by Nagata et al. [51]. Cells were subcultured once a week. For biolistic transformation, 1 ml of four-day old suspension cells (early logarithmic phase) was separated from the medium by sterile filtration through a 50 µm nylon mesh and then spread to a uniform cell layer on solid MS medium. Before biolistic transformation, cells were incubated in the dark at 24°C for 20 h. Following transformation, plates were incubated for 4 h in the dark and then subcultured in 3 ml of fresh liquid MS medium and further cultured at 24°C in a dark chamber with shaking at 80 rpm until microscopic observations. For microscopy, 100 µl of suspension culture was transferred onto cover slips fixed to the bottom of metal slides provided with a central opening (Φ 20 mm).

### Preparation of Whole Cell Extracts from Transiently Transformed BY2 Cells and Western Blot Analysis

Eight and 24 hours after transformation, BY2 cells were collected by centrifugation (5 min, 1000 *g*) and resuspended in extraction buffer (100 mM Tris pH 7.6; 0.5 sucrose, 0.1% (w/v) ascorbic acid, 0.1% (w/v) cystein-HCl) supplemented with EDTA-free protease inhibitor cocktail (Roche Diagnostics, Mannheim, Germany). The suspension was homogenized for 1 min at 25 Hz (Retsch MM 200) and mixed with Laemmli sample buffer (4∶1). Samples were denatured at 95°C for 5 min, centrifuged (5 min, 16000 *g*) and supernatants were analyzed by SDS-PAGE on 12% gels and electroblotted on a polyvinylidene difluoride membrane (Immobilon-P Transfer Membrane; 0,45 µm; Millipore). Blots were hybridized with mouse monoclonal anti-GFP 7.1 and 13.1 (Roche Diagnostics) 1∶1000, followed by goat anti-mouse IgG horseradish peroxidase-conjugated antibody (Sigma-Aldrich, St. Louis, MO) 1∶3000, and developed using an enhanced chemiluminescence system (ECL Western Blotting Substrate, Promega).

### MG132 Treatment

Eight hours after bombardment, BY2 cells were treated with 50 µM MG132. Following the addition of MG132 stock solution (10 mM, in DMSO) cells were further cultured at 24°C in a dark chamber with shaking at 80 rpm until microscopic observations. Control cell samples were untreated or treated with the equal concentration of DMSO.

### Imaging

Images were acquired by Axiovert 200 M fluorescence microscope operated by the AxioVision software 4.5 (Zeiss, Gottingen Germany).The Plan-Neofluar objective was used: 40x, 0.6NA, and the filter sets 13 for GFP (excitation BP 470/20 and emission BP 505–530) and 14 for RFP (excitation BP 510–560, emission LP 590) acquisitions. All images were recorded using an AxioCam camera (MrC, Zeiss, Göttingen, Germany). Z-stack multidimensional acquisitions were performed prior to co-localization analysis. 3D images were further deconvolved by using the Parallel iterative deconvolution method (20 iterations) provided by the Image J software (http://rsb.info.nih.gov/ij/). Further processing and assembly was performed by Image J and Illustrator CS4 (www.adobe.com).

### Co-localization and Statistical Analysis

To perform co-localization analysis (Fig. 4), Pearson’s coefficient (PC) was calculated using the Image J based plug-in JACoP v2.0 (Just Another Co-localization Plug-in), http://imagejdocu.tudor.lu/doku.php?id=plugin:analysis:jacop_2.0:just_another_colocalization_plugin:start). The obtained values were analyzed statistically by GraphPad Prism 5.0 software. The mean PC values, calculated separately from the population of 18 extracted cells, nuclei and nucleoli, were compared by using the Mann-Whitney non-parametric t test, which does not assume a Gaussian distribution of data. Error bars represent standard error of the mean (SEM).

### Bioinformatics Analysis

To predict subcellular localization we used the SubCellular Proteomic Database (http://suba.plantenergy.uwa.edu.au), which houses large scale proteomic and contains precompiled bioinformatic predictions. In order to compliment findings when using the database, we used YLoc ([52], www.multiloc.org/YLoc) and Plant-mPLoc ([53], www.csbio.sjtu.edu.cn/bioinf/plant-multi).

The NetNES ([54], www.cbs.dtu.dk/services/NetNES) was used to predict NES signals and the PredictProtein server ([45], www.predictprotein.org) was utilized for NLS prediction.
